# Liquid Content Detection In Transparent Containers: A Benchmark

**DOI:** 10.3390/s23156656

**Published:** 2023-07-25

**Authors:** You Wu, Hengzhou Ye, Yaqing Yang, Zhaodong Wang, Shuiwang Li

**Affiliations:** Guangxi Key Laboratory of Embedded Technology and Intelligent Information Processing, College of Information Science and Engineering, Guilin University of Technology, Guilin 541006, China

**Keywords:** liquid content estimation, transparent container detection, LCDTC dataset, benchmark

## Abstract

Various substances that possess liquid states include drinking water, various types of fuel, pharmaceuticals, and chemicals, which are indispensable in our daily lives. There are numerous real-world applications for liquid content detection in transparent containers, for example, service robots, pouring robots, security checks, industrial observation systems, etc. However, the majority of the existing methods either concentrate on transparent container detection or liquid height estimation; the former provides very limited information for more advanced computer vision tasks, whereas the latter is too demanding to generalize to open-world applications. In this paper, we propose a dataset for detecting liquid content in transparent containers (LCDTC), which presents an innovative task involving transparent container detection and liquid content estimation. The primary objective of this task is to obtain more information beyond the location of the container by additionally providing certain liquid content information which is easy to achieve with computer vision methods in various open-world applications. This task has potential applications in service robots, waste classification, security checks, and so on. The presented LCDTC dataset comprises 5916 images that have been extensively annotated through axis-aligned bounding boxes. We develop two baseline detectors, termed LCD-YOLOF and LCD-YOLOX, for the proposed dataset, based on two identity-preserved human posture detectors, i.e., IPH-YOLOF and IPH-YOLOX. By releasing LCDTC, we intend to stimulate more future works into the detection of liquid content in transparent containers and bring more focus to this challenging task.

## 1. Introduction

Plenty of existing work has investigated methods of detecting transparent containers [1,2,3], as well as liquid height or filled status estimation [4,5,6,7,8]. The former aims to locate all instances of transparent containers present in an image, usually involving both identifying the transparent containers and localizing the rectangular boundary surrounding each one. Nevertheless, in spite of vast applications in areas such as service robots [9,10], waste classification [11,12], security checks [13,14] and so on, it provides only very limited information, i.e., no more than presence and localization, about the detected transparent containers. The latter is intended to perceive or estimate the liquid (such as water, alcohol, and other beverages) inside transparent containers by predicting the height of the liquid levels or filled status within the containers. Although having a variety of applications in such areas as the food and chemical industries, biotechnology, and manufacturing applications, it is scenario-specific and application-dependent to a great degree, which may involve, for instance, different measuring methods (e.g., contact and non-invasive manners), different sensors (e.g., pressure, ultrasonic, radar, and camera sensors), and different precision requirements (e.g., precisely based on scale mark or roughly estimating the filled status) [15,16,17,18]. In this paper, we motivate and formulate a computer vision (CV) task that combines transparent container detection and liquid content estimation, which generalizes well to broader CV-based application scenarios and can be considered as a more informative upstream task for other CV-based applications.

Benefiting from great performances in deep learning, technologies for localizing and detecting transparent containers have progressed dramatically in recent years. For instance, Dhulekar et al. [1] used machine learning to solve the difficulties of bottle identification in designing a BRM (Bottle Recycling Machine), which achieved good accuracy with better detection speeds. Though using machine learning to identify bottles produced good results in this work, much depends on the images of the bottles used to train the algorithm. Do et al. [19] proposed the implementation of a plastic bottle surveillance system within the coastal area, utilizing Pascal Visual Object Classes (VOC) to annotate a dataset of 1125 images. For the segmentation of transparent objects, Xie et al. [20] recommend using the Trans10K dataset, which is a comprehensive dataset. More than 10,000 images from this dataset show real-world scenarios taking place in a natural environment, and Wang et al. [2] established a UAV-Bottle Dataset (UAV-BD) for detecting bottles in the wild using low-altitude unmanned aerial vehicles, which contains 34,791 bottle fully annotated images. Although these baseline detectors perform well on their dedicated datasets, they only detect the bottles for a specific scenario. Transparent container detection poses a significant challenge due to the complexity of backgrounds in real-world environments, which may include kitchens, living rooms, the outdoors, and other intricate settings. Moreover, transparent container detection can only provide the essential data elements needed by computer vision applications, i.e., the locations of the container in a scene. For more complex computer vision work, this limited information is insufficient. For example, in the restaurant industry, liquid content detection in transparent containers such as beverage glasses and liquor bottles is crucial for the application of service robots [9,10], scene understanding [21], and security checks [13,14].

Due to its essence of being application-dependent, liquid content estimation has diverse methodologies. For instance, in the application scenarios where the containers are opaque, knowledge about the container and the liquid is usually necessary and very helpful. For example, Schenck et al. proposed to heat the liquid to perceive transparent liquids in an opaque container and utilized a thermal image to acquire the liquid’s content estimate in [22]. Dong et al. [9] developed a method for dynamically estimating liquid height in a cup by using the relation between the angle of the pouring container and poured volume. In this paper, we concentrate on estimating the liquid content of transparent containers instead. There has been a lot of work dedicated to this problem before. For instance, Li et al. [23] proposed to combine the style transfer method with a segmentation network [24] to sense transparent liquids (such as water) in transparent containers. The application of contrastive learning to convert a transparent liquid image into a colorful liquid image enables the perception of clear liquids without requiring further operations, thus easing the constraints on the operational domain. Kennedy et al. [25] suggested a method of detecting fluids in transparent containers by combining weight and vision. This approach involved pouring the transparent liquid onto a checkerboard background and then recording the weight to predict it. Although these approaches are able to provide high precision in measuring liquid level (volume) in transparent containers, the process is complicated and not practical in many real-life applications. In fact, we human beings often make decisions based on very crude estimates of liquid content in transparent containers with our eyes in daily life. Mimicking and implementing such a visual function is no doubt very useful to robots and other intelligent systems, especially equipped with visible light cameras, when crude estimates about the liquid content in transparent containers is enough, which motivates our work here.

**Figure 1:**
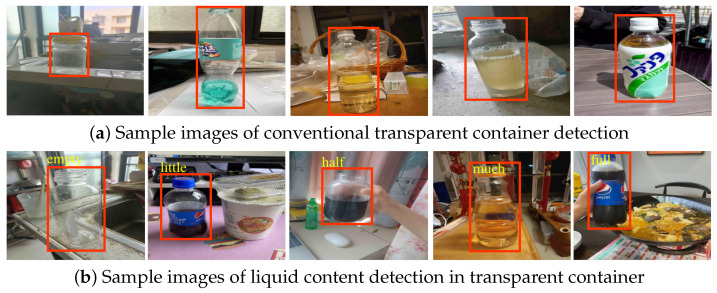
The comparation of traditional transparent container detection (**a**) and our proposed liquid content detection in a transparent container (**b**). The former pays attention to the identification and localization of the containers, but our proposed method can achieve additional information by introducing an extra state head, i.e., ‘empty’, ‘little’, ‘half’, ‘much’, and ‘full’ are marked in (**b**) from left to right, respectively.

In this paper, we dedicate ourselves to a new CV task which is a combination of transparent container detection and liquid content estimation. Specifically, we focus on crude estimations of liquid content inside transparent containers and accurate localization of the containers in RGB images in which the liquid content is crudely divided into five types, i.e., ‘empty’, ‘little’, ‘half’, ‘much’, and ‘full’. An illustration of the distinction between traditional transparent container detection and our liquid content detection in transparent containers is shown in Figure 1. As additional information about liquid content is provided and a variety of daily-life transparent containers are taken into account, this task has wide potential applications in service robots [9,10], scene understanding [21], security checks [13,14], waste classification [11,12], and so on. We think that this work might have far-reaching implications in computer vision perception, analysis, and interpretation, and that it may lead to further investigations of new detection tasks beyond identification and localization. This task is called Detection of Liquid Content in Transparent Container, for which we present a dataset to facilitate future studies, called the LCDTC dataset. The LCDTC dataset is made up of transparent containers, containing 5916 images inclusive of axis-aligned bounding boxes and the liquid content present within the containers.

### Contribution

**Figure 2:**
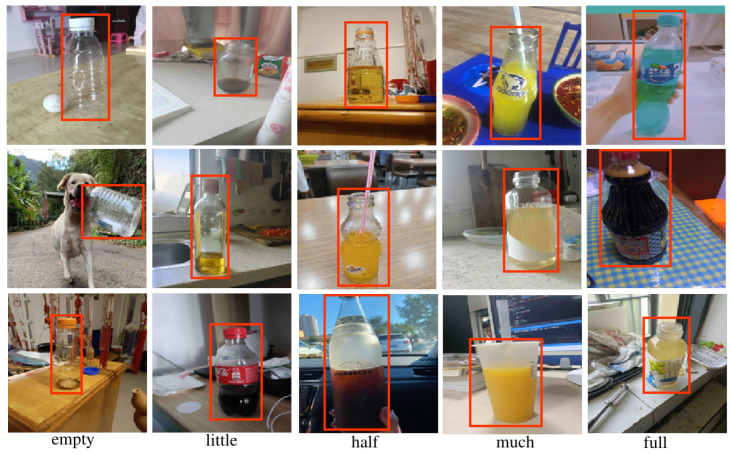
Examples of transparent containers with five liquid content (i.e., ‘empty’, ‘little’, ‘half’, ‘much’, and ‘full’ from left to right) are shown in the proposed LCDTC dataset. Red boundary boxes serve as an identifier for the objects.

In this work, we present a dedicated dataset for detecting liquid content in transparent containers, which contains 5916 images inclusive of axis-aligned bounding boxes and the liquid content present within the containers. By using this dataset, we motivate and formulate a CV task that combines transparent container detection and liquid content estimation, called Liquid Content Detection in Transparent Container. We show 15 sample images from the LCDTC dataset in Figure 2. Moreover, we have established two baseline detectors, termed LCD-YOLOF and LCD-YOLOX, which are based on two identity-preserved human posture detectors, i.e., IPH-YOLOF and IPH-YOLOX. We use these baseline detectors to evaluate the performance of this task. Moreover, they will serve as useful comparisons for future LCDTC investigations.

Our contribution can be summarized as follows:

- We have proposed a challenging task which combines transparent container detection and liquid content estimation. This task encourages more advanced applications while offering a new perspective on transparent container detection.
- We present the LCDTC dataset, the first benchmark for identifying the liquid content in a transparent container.
- Based on two identity-preserved human posture detectors, we have created two baseline detectors by introducing Triplet Attention [26] and CrossFormer [27] to the original model for better performance.

## 2. Related Works

### 2.1. Traditional Methods for Detecting Transparent Containers

Detecting transparent containers has proven to be a challenging task due to their inherent lack of distinct visual features. In traditional transparent container detection, Klanl [28] proposed a method for transparent object detection and reconstruction by leveraging the principle that such objects absorb light at specific wavelengths. Their approach involves employing a conventional infrared emitter alongside the intensity sensor of a time-of-flight camera for structure reconstruction. Nevertheless, the performance of this approach is limited, with a success rate of approximately 55%, as the sole cue utilized for the identification of transparent objects is infrared light intensity. Lei et al. [29] effectively utilized light detection and ranging (LIDAR) data to accurately segment transparent objects. They employed a technique that involved identifying candidate areas of transparent objects from a RGB image using highlight spots and then applying the GrabCut segmentation algorithm (developed by Rother et al. [30]) to a depth image and a laser reflectance intensity image to finalize the segmentation. This method demonstrated reliable detection of bottle-shaped transparent objects at close distances using a visual sensor. Osadchy et al. [31] made use of the specular highlights functionality, which necessitates a light source and is essential for identifying glass objects from other things. McHenry et al. [32] thoroughly evaluated various characteristics, comprising similarities in color, extent of blurring, consistency of overlay, and perturbations in texture, alongside significant highlights for the efficient identification of transparent objects. On a dataset containing four transparent objects, Fritz [33] used an additive model of latent factors along with a combined method of SIFT and Latent Dirichlet Allocation (LDA) to calculate the appearance of a transparent local patch. This method successfully identifies transparent objects against a variety of backgrounds. Despite the historical value of traditional methodologies in advancing the field of transparent container development, their performance pales in comparison to the more contemporary, deep learning-based approaches which have achieved great success in recent times.

### 2.2. Deep Learning Methods for Detecting Transparent Containers

Currently, most researchers are keen to apply deep learning networks for transparent container detection thanks to deep learning’s amazing skill in extracting discriminative features. For example, the application of Region with Convolutional Neural Network (R-CNN) was leveraged by Lai et al. [34] to facilitate the recognition of transparent objects within color images. The R-CNN method employs selective search [35] to extract interested region proposals [36]. The selective search algorithm’s performance is increased by taking into account the highlight and color similarity features of transparent objects in order to eliminate some region proposals that are not transparent. Khaing et al. [37] used one of the Convolutional Neural Networks, called Single Shot Multibox Detector (SSD) [38], to detect transparent objects in images, which achieved accurate and considerable results. An issue in SSD is the potential misclassification of non-transparent objects with a similar shape to transparent objects, resulting in non-transparent objects being incorrectly identified as transparent by SSD. Consequently, a second training procedure is performed on the neural network using a negative training dataset to learn the properties that distinguish transparent from non-transparent objects of the same shape. Seib et al. [39] proposed a method to exploit sensor failures in depth images for transparent object localization using convolutional networks. In view of the fact that methods based on deep learning typically suffer from boundary-related imbalances, which can adversely affect the extent of their generalization, Cao et al. [40] proposed an innovative data augmentation approach, named FakeMix, that is designed to address the issue of boundary-related imbalances. Moreover, they introduced AdaptiveASPP, an improved iteration of ASPP [41], which dynamically captures cross-modality and multi-scale features. Benefiting from FakeMix and AdaptiveASPP, their FANet model has been demonstrated to be quite effective. In recent years, researchers have been exploring the use of YOLO (You Only Look Once) in detecting transparent containers, showing promising and encouraging results. For example, in identifying plastic bottles (e.g., those that are mostly transparent), the YOLO algorithm with the COCO dataset produced quick and precise results [42,43]. Akbar et al. [44] conducted research on the object recognition of bottles using the YOLOv3 algorithm and applied it through a mobile phone camera, which achieved good detection performance. Ju et al. [45] proposed a novel method, namely NMYOLO, which builds on the YOLOv4 [46] model to effectively address the challenge of identifying medical infusion containers in complex environmental scenarios. Specifically, the proposed model is augmented by incorporating ASFF [47] and CA [48] and introducing improved cross-stage partial-spatial pyramid pooling (CSP-SPP) to replace origin spatial pyramid pooling (SPP). Furthermore, EIOU [49] is utilized in order to enhance the stability of the model. These improvements make NMYOLO have a better performance.

The YOLO algorithm is a one-stage object detection model that has gained significant attention in the field of computer vision. Using a single neural network, YOLO regards object detection as a regression task with spatially separated bounding boxes and related class probabilities. By predicting the entire image in one go, YOLO significantly accelerates object detection and offers high accuracy. The YOLO series and its variants have proven very successful in the field of object detection. In this paper, we employ the two identity-preserved human posture detectors to establish our baseline detectors for identifying liquid content in transparent containers, whose network architecture is inherited from that of the two most current YOLO variants.

### 2.3. Liquid Content Estimation

Liquid content estimation is an important task in various industries, including food and beverage, pharmaceuticals, and chemical processing. The methodologies can be divided according to whether the containers are opaque or transparent. Opaque containers, such as metal cans or plastic bottles, present a relatively straightforward challenge for liquid content estimation. This is because the contents of the container are not visible, so generally other methods instead of vision-based approaches must be employed. For example, Aoyagi et al. [5] proposed a method to observe the liquid level in an opaque metal container by dynamic heat conduction. Specifically, this method requires rapid heating of the container via a flash, coupled with infrared monitoring of changes in surface temperature to predict the liquid level in an opaque metal vessel. It was proved to be effective in predicting the liquid level in vessels made out of copper, brass, aluminum, and polyvinyl chloride. By utilizing an RGB-D camera, Do et al. [4] proposed a pioneering probabilistic way to predict the liquid level in a container. This process involved utilizing several point clouds and images captured at various viewing angles of a cup containing a liquid, enabling the estimation of its liquid level from the acquired information.

Transparent containers, on the other hand, present a unique challenge for liquid content estimation. The contents of the container are visible, but the reflective and refractive properties of the container and the liquid can make accurate estimation difficult. Nonetheless, vision-based methods are frequently preferred due to several reasons. First, vision-based methods allow for non-invasive and non-contact measurements, which is particularly important in industries such as pharmaceuticals and food and beverage where contamination of the product must be avoided. Second, the potential cost savings in certain applications make them an attractive option. Third, vision-based methods can be highly automated, reducing the need for human intervention and increasing efficiency. For instance, Li et al. [23] proposed to combine the style transfer method with a segmentation network [24] to sense transparent liquids (such as water) in transparent containers. It can accurately identify the transparent liquid in the container and then provide assistance in estimating the liquid level height and volume of the transparent liquid. Feng [50] proposed a novel approach to predict the location of liquid surfaces in transparent containers that is grounded on visual analysis. Specifically, following binarization, the images were segmented using the Shen algorithm [51] and the projection statistic method, smoothing the horizontal projection image, splitting the smooth after the projection line, and predicting the liquid level position [52]. By analyzing contextual cues from neighboring objects, Mottaghi et al. [53] proposed a method based on deep learning for estimating the volume of containers and their contents. According to their experimental evaluations, the inclusion of contextual cues yields better performance in the estimation of container volume and content. Additionally, by combining convolutional and recurrent neural networks, it can predict how liquid contents inside containers will behave in response to the container tilting, which is crucial for completing the pouring task. In their study, they divided the space of volumes into 10 classes, where the maximum volumes in each class were 50, 100, 200, 300, 500, 750, 1000, 2000, 3000, and *∞*. The unit of measurement was the milliliter (mL). Liquid content for the transparent container was based on one of five categories, namely, 0% (empty), 33%, 50%, 66%, or 100%.

These methods can measure liquid level (volume) in transparent containers with a high degree of accuracy, but the procedure is cumbersome and impractical in many real-world situations. In fact, we humans frequently base our daily decisions on very rough estimations of the liquid content of transparent containers using our eyes. Our work is motivated by the fact that mimicking and implementing such a visual function is undoubtedly very helpful for robots and other intelligent systems, especially those equipped with visible light cameras, when rough estimates of the liquid content in transparent containers is sufficient. In this work, we propose an innovative task which is a combination of transparent containers detection and liquid content estimation. Our task focuses on crude estimation of liquid content inside transparent containers and accurate localization of the containers in RGB images, in which the liquid content is crudely divided into five types, i.e., ‘empty’, ‘little’, ‘half’, ‘much’, and ‘full’.

## 3. Benchmark for Liquid Content Detection in Transparent Containers

The technical details about the methods and pipelines we use to construct LCDTC are described in this section. In building LCDTC, which will be covered in more detail later, we cover a wide range of transparent containers and offer the manual annotations for each image.

### 3.1. LCDTC Collection

Collecting a large and diverse dataset of images is crucial for training and evaluating machine learning models for liquid content detection in transparent containers. However, it can be challenging to find images containing transparent containers with various backgrounds and liquid content states. To address this issue, we decided to combine internet-sourced images with images captured by ourselves, which allowed us to create a more diverse and balanced dataset. Specifically, we collect approximately 2000 images from the Internet, with nearly 4000 images captured by experts (i.e., a student engaged in object detection) using cameras equipped in cellphones (i.e., vivoS 7, realme q3 pro). To broaden the variety of the dataset, these images were collected from a variety of scenarios in everyday life, including settings such as the living room, kitchens, restrooms, outdoor areas, restaurants, and so on. In addition, the images are distinguished by a significant variation in viewing conditions, encompassing object size, background, liquid content, etc. Considering that there may be ambiguities between two adjacent liquid statuses, e.g., little and much, we intentionally excluded those images if they bother us to ponder how to label their liquid status. Moreover, manually labeling and predicting the liquid content in transparent containers is also challenged by unclear or blurry images. These images are also omitted from our dataset. The LCDTC dataset consists of 5916 well-annotated images and is divided into two distinct sets: a train set and a test set, including 4437 and 1479 images, respectively. Moreover, we tried to make different liquid content states distribute equally as much as possible to reduce the influence of unbalance samples in the train data. Moreover, we considered the five most typical liquid contents in containers in daily life when building LCDTC, i.e., ‘empty’, ‘little’, ‘half’, ‘much’, and ‘full’. In Figure 2, we show several representative images of each state in the LCDTC dataset.

### 3.2. Annotation

In this section, we describe the annotation process of our dataset from three aspects: category, boundary box, and liquid content state. The following is a detailed introduction.

- **Category:** transparent container.
- **Bounding box:** a bounding box centered on the image’s visible transparent containers with axis alignment.
- **Liquid content state:** one of ‘empty’, ‘little’, ‘half’, ‘much’, and ‘full’.

Adhering to standards is essential when it comes to annotation, as it ensures that the annotations are consistent and accurate. The annotation guide [54,55] provides a set of guidelines and instructions that annotators should follow when labeling data: firstly, the expert (i.e., a student engaged in object detection) should maintain consistency in image annotations, particularly with regard to class definitions, bounding box placement, and the accurate depiction of views and truncation. Secondly, it is crucial that all object instances are labeled precisely and that certain measures are taken to minimize any potential errors in this process. To successfully complete the annotation work, the manual annotation procedure, visual examination, and box refining approaches are all carefully carried out. In the first step, for a given image, an expert will initially label it following the above guidelines. In the second step, we will submit the annotated dataset to the inspection team for review, including checking for omitted objects to ensure exhaustive labelling. The annotation errors will be refined in the third stage, which will be sent back to the initial annotation for refinement. With this three stage strategy, the dataset can be ensured that the annotation of objects it contains are of high quality. See Figure 2 for examples of box annotations for LCDTC.

### 3.3. Dataset Statistics

**Figure 3:**
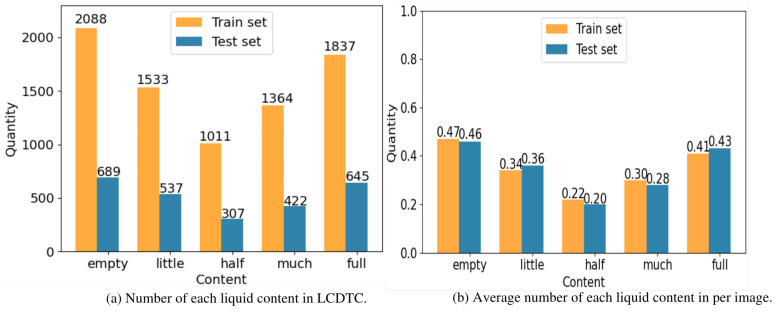
(**a**) shows the number of each liquid content in transparent container in the train set and test set on the LCDTC and (**b**) shows the average number of each liquid content per image in the train set and test set on the LCDTC.

**Figure 4:**
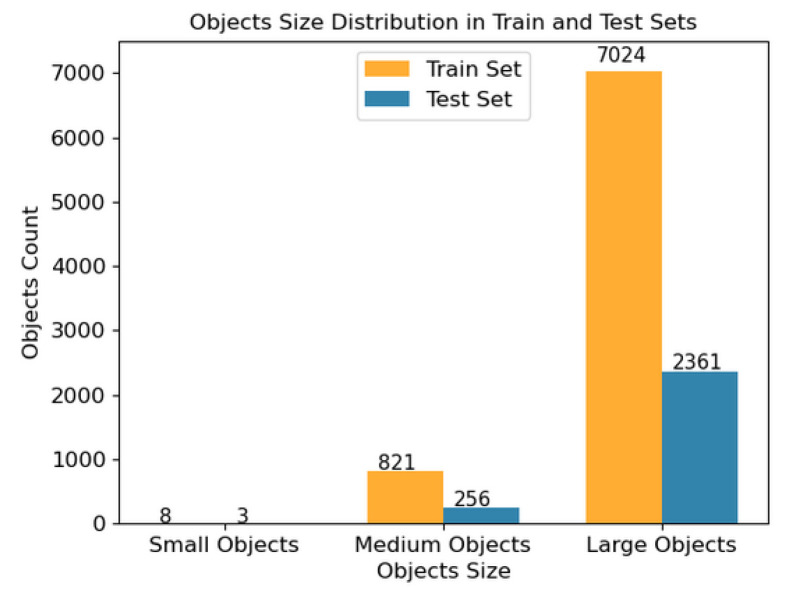
The distribution of object sizes for small, medium, and large in the LCDTC dataset.

We divide the LCDTC dataset into two primary subsets, i.e., the train set and the test set, to facilitate training and evaluation. The allocation ratio between the two subsets is 7:3. The LCDTC dataset includes a total of 5916 well-annotated images, each containing one or more transparent containers. In total, we have annotated 10,453 transparent containers, providing accurate information about their liquid content. In Figure 3, we show the statistics of the LCDTC dataset by histogram. Figure 3a depicts the number of each liquid content in the LCDTC dataset’s train set and test set, whereas Figure 3b depicts the average number of each liquid content per image in the LCDTC dataset’s train set and test set. As can be seen, the distribution of the five liquid statuses in LCDTC varies widely, suggesting an imbalanced distribution of liquid statuses across the dataset. This reflects the true frequency of encountering such objects in the real-life scenarios where we collected the dataset. Although concerns might be raised about the potential sample imbalance problem in the dataset, our experimental results effectively alleviate these concerns. Moreover, according to the defined criteria in the COCO dataset, objects with a pixel area smaller than 32 × 32 are classified as small target objects, objects with a pixel area ranging between 32 × 32 and 96 × 96 are considered medium target objects, while objects with a pixel area larger than 96 × 96 are categorized as large target objects. However, it is challenging to determine the state of liquids in transparent containers with small target sizes, as liquids lack a fixed shape and are typically transparent. As a result, our dataset does not currently include objects with smaller target sizes. Figure 4 displays detailed sample classification statistics for small, medium, and large in the LCDTC dataset.

## 4. Baseline Detectors for Liquid Content in Transparent Containers

In order to promote the development of liquid content detection in transparent containers, we propose two baseline detectors based on IPH-YOLOF and IPH-YOLOX which were proposed for identity-preserved human posture detection [56]. These two detectors are established on two state-of-the-art YOLO variants (YOLOX [57] and YOLOF [58]) by introducing an additional classification head to each original model to predict each person’s posture. We adapt these two detectors for our task. In addition, we apply an effective attention to the extra head, called triplet attention [26], to improve the performance of the liquid content estimation. Notably, IPH-YOLOX relies on the capacity to fuse multiple-level features due to its use of PANet [59], an enhancement based on FPN, as the neck sub-network. To improve the performance of IPH-YOLOX, we use the CrossFormer [27], which is built on the interactions among features of different scales, as the backbone network to replace the origin one. However, IPH-YOLOF utilizes solely the highest level features for detection, which restricts the interactions among the features of varied scales. Therefore, we do not modify the original backbone network in IPH-YOLOF. The proposed baseline detectors are called LCD-YOLOF and LCD-YOLOX, respectively, which are described in detail in the following.

### 4.1. LCD-YOLOF

**Figure 5:**
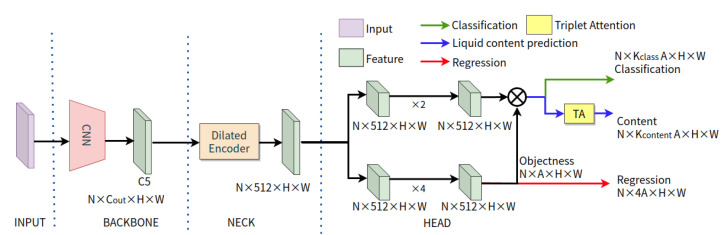
The network architecture of our baseline LCD-YOLOF detector, which is inherited from that of IPH-YOLOF [56]. The liquid content prediction head with triplet attention is the difference to the original IPH-YOLOF.

In Figure 5, we show that the network architecture of the LCD-YOLOF employs the classical ResNet50 [60] as the backbone network, which is pre-trained on the widely-used ImageNet [61] dataset. The C5 stands for the backbone’s output feature representation, which has 2048 channels and a downsampling multiplicity of 32/16. The neck sub-network’s dilated encoder receives these features and holds responsibility for executing the encoding process. The classification head and the regression head are two concurrent task-specific heads that make up the head of the original YOLOF. Similar to the IPH-YOLOF model, we also introduce a new classification head to predict the state of the liquid content in transparent containers. To enhance the performance of liquid content estimation, we add an effective attention mechanism in this extra head, known as triplet attention [26]. The triplet attention is designed to capture inter-dimensional dependencies in the input tensor by utilizing a rotation of the tensor and subsequent residual transformations. For more details, please refer to Section 4.3. The following is the definition of the total loss of training LCD-YOLOF:

(1) $$L_{total}=L_{cls}+L_{reg}+\lambda L_{content}.$$

The total loss is comprised of the losses related with classification, regression, and liquid content prediction, denoted by $L_{cls}$, $L_{reg}$, and $L_{content}$, respectively. λ is a constant which represents the weight coefficient of the loss for the liquid content prediction head. The definitions of these losses are provided below [56,62],

$$L_{\mathrm{cls}}=\frac{1}{N_{\mathrm{pos}}}\sum_{n=0}^{N_{\mathrm{pos}}}FL\left(y_{\mathrm{cls}}^{n},p_{\mathrm{cls}}^{n}⊗p_{\mathrm{obj}}^{n}\right),$$

$$L_{\mathrm{reg}}=\frac{1}{N_{\mathrm{pos}}}\sum_{n=0}^{N_{\mathrm{pos}}}\left({\mathrm{error}}_{L_{1}}\left(b_{t}^{n}-b_{p}^{n}\right)\right),$$

(2) $$L_{\mathrm{content}}=\frac{1}{N_{\mathrm{pos}}}\sum_{n=0}^{N_{\mathrm{pos}}}FL\left(y_{\mathrm{content}}^{n},p_{\mathrm{content}}^{n}⊗p_{\mathrm{obj}}^{n}\right),$$

where the variables $y_{\mathrm{cls}}^{n}$ and $y_{\mathrm{content}}^{n}$ indicate the ground truth for classification and liquid content estimation, respectively. The variables $p_{cls}^{n}$, $p_{content}^{n}$, and $p_{obj}^{n}$ denote the predictions of classification, state of liquid content, and boxes (i.e., whether the box contain any object), respectively. *FL* (·) and $smooth_{L_{1}}$ indicate the focal loss [63] and the $smooth_{L_{1}}$ loss functions, respectively. The focal loss function is a commonly used technique for addressing the issue of imbalance between difficult and easy samples in machine learning models. Specifically, it works by increasing the weight of the small number of target categories and misclassified samples. This approach helps the model to focus more on these difficult samples during training, which can lead to improved performance and accuracy. On the other hand, the $smooth_{L_{1}}$ loss function is often preferred over traditional $L_{1}$ and $L_{2}$ loss functions because it is less sensitive to outliers. Moreover, $smooth_{L_{1}}$ loss helps to prevent the issue of gradient explosion during training by controlling the magnitude of the gradient. This can lead to more stable and consistent training. ${⊗}_{p}$ represents the scalar product. $N_{pos}$ is the number of positive anchors. The variables $b_{t}^{n}$, and $b_{p}^{n}$ denote the ground truth-bounding box and the predicted bounding box, respectively.

### 4.2. LCD-YOLOX

**Figure 6:**
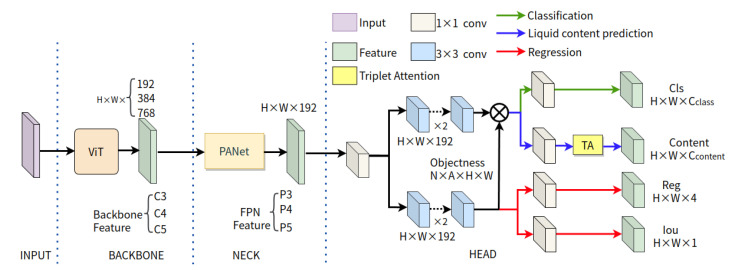
The network architecture of our baseline LCD-YOLOX detector, which is inherited from that of IPH-YOLOX [56]. The CrossFomer backbone and liquid content prediction head with triplet attention are the difference to the original IPH-YOLOX.

In Figure 6, we show the network architecture of LCD-YOLOX. To enhance the performance of this baseline detector, we use the CrossFormer [27], which is based on a cross-scale attention module, as the backbone network to replace the origin one (e.g., CSPDarkNet and the Spatial Pyramid Pooling(SPP) [64]). According to research by [65,66,67], CrossFormer makes use of a pyramid structure to separate the transformer model into four distinct stages. Each stage consists of a cross-scale embedding layer (CEL) and several CrossFormer blocks. Specifically, the Cross-scale Embedding Layer (CEL) has cross-scale features, and Long Short Distance Attention (LSDA) can facilitate cost-efficiency in the self-attention module, without undermining the integrity of small-scale or large-scale features. For more details, please refer to [27]. The C3, C4, and C5 indicate the backbone’s output features, with corresponding channels of 192, 384, and 768, respectively. The neck sub-network’s PANet [59] receives these features and holds responsibility for executing the encoding process. This network utilizes a bottom-up path to combine deep features with shallow ones, followed by the integration of deep features with a top-down path. The classification head and the regression head are two concurrent task-specific heads that make up the head of the original YOLOX. In our proposed LCD-YOLOX model, we also introduce a new classification head to predict the state of liquid content in transparent containers, which has the same architecture as the one introduced for LCD-YOLOF. The following is the definition of the total loss of training LCD-YOLOX:

(3) $$L_{total}=L_{cls}+L_{reg}+L_{obj}+\lambda L_{content}.$$

The total loss is made up of the losses related with classification, regression, the confidence of boxes, and liquid content prediction, denoted by $L_{cls}$, $L_{reg}$, $L_{obj}$, and $L_{content}$, respectively. λ is a constant which denotes the weight coefficient of loss for the liquid content prediction head. The definitions of these losses are as follows [56,62]:

$$L_{cls}=\frac{-1}{N_{pos}}\sum_{n=1}^{N_{\mathrm{pos}}}y_{cls}^{n}\ln\left(\sigma \left(p_{cls}^{n}\right)\right),$$

$$L_{\mathrm{content}}=\frac{-1}{N_{\mathrm{pos}}}\sum_{n=1}^{N_{pos}}y_{\mathrm{content}}^{n}\ln\left(\sigma \left(p_{\mathrm{content}}^{n}\right)\right),$$

$$L_{obj}=\frac{-1}{N_{pos}}\sum_{n=1}^{N_{pos}}y_{obj}^{n}\ln\left(\sigma \left(p_{obj}^{n}\right)\right),$$

(4) $$L_{reg}=\frac{1}{N_{pos}}\sum_{n=1}^{N_{pos}}\left(1-\mathrm{IOU}\left(b_{t}^{n},b_{p}^{n}\right)\right).$$

where the variables $y_{cls}^{n}$, $y_{content}^{n}$, and $y_{obj}^{n}$ denote the ground truth for classification, liquid content, and boxes, respectively. $p_{cls}^{n}$, $p_{content}^{n}$, and $p_{obj}^{n}$ indicate the predictions for classification, state of liquid content, and boxes, respectively. The softmax activation and *IOU* loss functions are denoted by σ and *IOU*(·), respectively. The IOU metric measures the gap between the ground truth bounding box and the predicted bounding box. Notably, IOU has the property of scale invariance. $N_{pos}$ is the number of positive anchors. The variables $b_{t}^{n}$, and $b_{p}^{n}$ denote the ground truth-bounding box and the predicted bounding box, respectively.

### 4.3. Convolutional Triplet Attention Module (CTAM)

**Figure 7:**
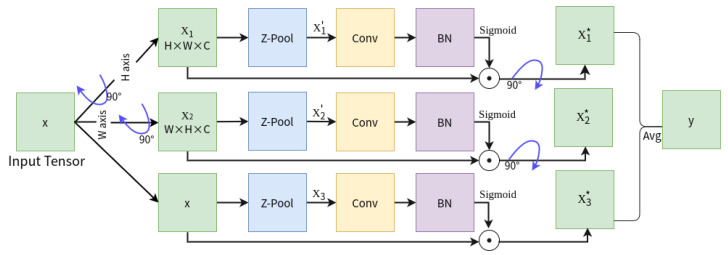
The architecture of the Convolutional Triple Attention Module (CTAM) is depicted in the illustration. In these branches, the symbol * is used to mark the variable obtained by multiplying broadcast elements in the corresponding branch. The symbol ⊙ denotes broadcast element-wise multiplication.

The Convolutional Triplet Attention Module (CTAM) is a lightweight yet effective attention module. In this paper, we deployed it to the two improved baseline detectors’ extra prediction heads for predicting the liquid content of transparent containers to further enhance the performance of liquid content detection. It is designed to capture cross-dimensional interactions in the input tensor by utilizing a rotation of the tensor and subsequent residual transformations to establish inter-dimensional correlations. A large number of feature representations are produced by the calculation of attention weights, leading to the generation of a refined tensor with the same form as the original input. We show the detailed architecture of the CTAM in Figure 7. As shown in the figure, three parallel branches make up the CTAM model, with two dedicated to capturing the interaction between the channel dimension C and one of the spatial dimensions, i.e., H or W, and another branch is used for the development of spatial attention. The output of all three branches is obtained using a straightforward averaging method. Specifically, CTAM receives an input tensor x $\in R^{C\times H\times W}$, where C denotes the number of channels and H and W represent the height and width of the spatial feature maps, respectively. At the beginning, this tensor is delivered to all three branches. The first branch experiences an interaction between the height and the channel dimension. Subsequently, rotation of x by 90 counterclockwise occurs along the H axis, resulting in the formation of $x_{1}$ with a shape of (W×H×C), which undergoes minimization via Z-pool to $x_{1}^{\prime }$ with dimensions of (2×H×C). The convoluted layer is then applied to $x_{1}^{\prime }$, followed by a batch normalization layer. Furthermore, the attention weights are obtained by transmitting them to the sigmoid activation layer. In order to preserve the fundamental structure of the input, these weights are applied to $x_{1}$ and subjected to a 90 clockwise rotation about the H axis. $x_{1}^{*}$ is the tensor generated at the completion of the first branch.

Similar to the first branch, the refined $x_{2}^{*}$ is obtained in the second branch by rotating x 90 counterclockwise along the W axis using the same methodology as in the first branch. In the final branch, the Z-pool reduces the channels of the input tensor x to two and produces $x_{3}$ having the shape (2×H×W), which is then processed by a convolution layer. Then, it proceeds successively through a batch normalization layer. The output generates an attention weight with the shape (1×H×W) via the sigmoid activation layer; the tensor of the last branch generated at the end is defined as $x_{3}^{*}$. The three branches’ produced data were simply averaged to provide the refined shape tensor (C×H×W). To summarize, the following equation shows how the refinement tensor y is created from the three branches for an input tensor x $\in R^{C\times H\times W}$:

(5) $$y=\frac{x_{1}^{*}\omega_{1}+x_{2}^{*}\omega_{2}+x_{3}^{*}\omega_{3}}{3}$$

The three cross-dimensional attention weights calculated in the triplet attention are denoted by the variables $\omega_{1},\omega_{2}$ and $\omega_{3}$.

## 5. Evaluation

### 5.1. Evaluation Metrics

In the experiment, we evaluate the performance of the two baseline detectors by using AP (i.e., Average Precision) and mAP (i.e., mean Average Precision). IOU (Intersection of Union) is used to measure the error between the predicted bbox and the ground truth bbox. AP evaluates the average precision for one specific object, and its value is calculated by finding the area under the precision-recall curve in relation to the coordinate axes. Meanwhile, Average Precision (mAP) evaluates the performance of the algorithm across multiple targets or classes. It is obtained by calculating the average of all the individual AP values for each target. Precision is the ratio of the number of true positive detections to the total number of detections that the algorithm has predicted as being positive, defined by Precision=TP/(TP+FP), where TP is the number of true positive detections and FP is the number of false positive detections. Recall, on the other hand, measures the completeness of the detection algorithm. It is defined as the ratio of the number of true positive detections to the total number of objects in the image: Recall=TP/(TP+FN), where FN is the number of false negative detections. Please see [54] for a more thorough description.

According to the COCO evaluation metric [55], 0.5, 0.75, and 0.5 to 0.95 are set as an IoU threshold to check whether the predicted bounding box is valid. IoU values of 0.5 and 0.75 are denoted by AP@0.5 and AP@0.75, respectively; when IoU is between 0.5 and 0.95, with step size 0.05, the corresponding AP is denoted by AP@[0.50:0.05:0.95]. The COCO mAP metric was used to assess the detector’s performance in identifying liquid content in transparent containers. Precision in classical object detection simply predicts the accuracy of the target category. However, we propose this new detection task, which is composed of transparent container detection and liquid content estimation. In order to evaluate the proposed detectors’ performance on our task, it is necessary for the precision metric to incorporate the simultaneous prediction of both category and liquid content. Following [56,62], in this paper, $AP_{c}$, $AP_{t}$, and $AP_{ct}$ are denoted as the precision metric for the prediction of the object’s category, state of liquid content, and the composite of the two, respectively, and by introducing a additional prefix ’m’, denoting the mean AP, i.e., mAP.

### 5.2. Evaluation Results

**Overall Performance.** The two baseline detectors we propose, i.e., LCD-YOLOF and LCD-YOLOX, are comprehensively evaluated on the LCDTC dataset. Table 1 displays the evaluation results with the precision metrics $AP_{c}$, $AP_{t}$, and $AP_{ct}$ as defined in Section 5.1. It is evident that LCD-YOLOX outperforms LCD-YOLOF, given that all of its Average Precisions (APs) are higher than those of LCD-YOLOF. Remarkably, LCD-YOLOX significantly surpasses LCD-YOLOF on $AP_{t}@0.5$, $AP_{t}@0.75$, and $mAP_{t}$, with gains of 5.4%, 5.4%, and 6.4%, respectively. Moreover, evaluation results suggest that the average precision of detecting transparent containers is greater than that of detecting liquid content for both detectors. Specifically, it can be observed that the differences between $mAP_{c}$ and $mAP_{t}$ are all above 17%, with the biggest gap of 19.1% appearing in LCD-YOLOF. These results suggest that distinguishing a transparent container from its environment is comparatively simpler than determining its precise liquid content state. The latter encounters greater intra-class differences, including the liquid’s resemblance to the background and absence of a consistent shape. Recognizing the challenge this task presents, we anticipate that our research will inspire additional investigators to delve into the investigation of liquid content detection in transparent containers.

**Performance on per liquid content state.** We assess the effectiveness of the two proposed baseline detectors on each liquid content state. This allows us to get a better understanding and analysis of liquid content detection in transparent containers. The $mAP_{ct}$ of the two baseline detectors is shown in Table 2. As can be seen, all $mAP_{ct}$s of LCD-YOLOX are larger than 50%, except in the ‘half’ content state, while LCD-YOLOF has only one $mAP_{ct}$ greater than 50% in the ‘little’ content state. Moreover, it is clear that all the detectors exhibit the best performance in ‘little’, followed by ‘empty’, while the $mAP_{ct}$ of ‘half’, ‘much’, and ‘full’ have lower performances. The following factors can be attributed to this observation: (1) the ‘little’ content state has a relatively clear texture features in the image; (2) the ‘empty’ accounts for one fourth of the proportion in training data, which is the largest amount, with which the model is easier to learning more robust discriminative cues; (3) there are more intra-class variations in ‘half’ and ‘much’ than in ‘empty’ and ‘full’ due to liquid’s lack of a fixed shape. More specifically, despite the training data not being the largest, the ‘little’ liquid content state usually exhibits relatively clear texture features and smaller liquid content state variations, which makes its detection relatively easier. On the other hand, while ‘empty’ and ‘full’ possess the largest and second largest number of training data, respectively, the detectors tend to confuse these two states when the liquid inside the container is transparent. In our future work, we will consider these factors to develop more effective detectors for detecting liquid content in transparent containers in RGB images.

**Figure 8:**
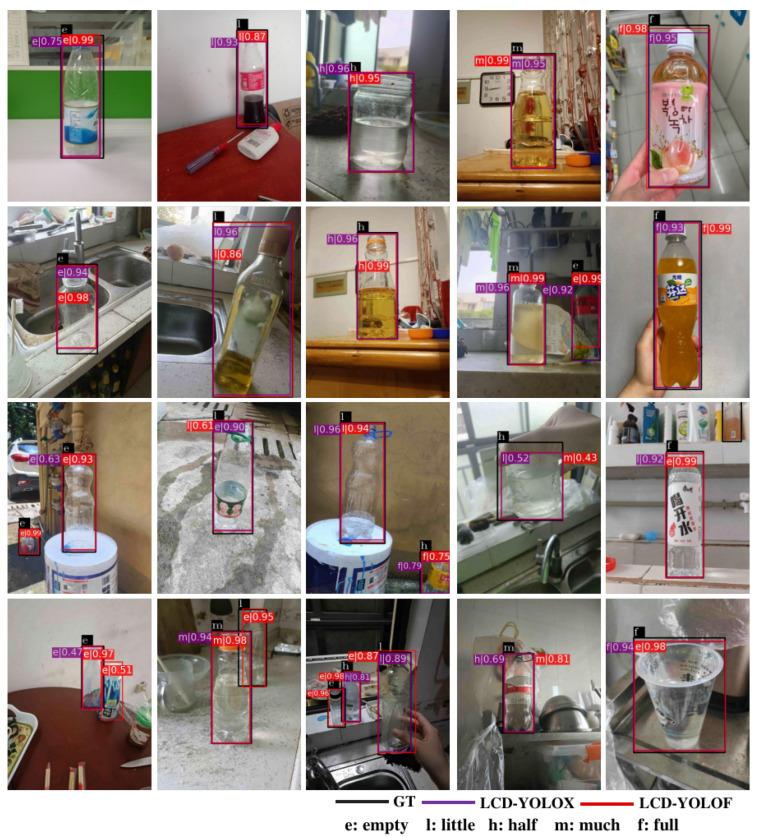
Qualitative evaluation on 20 samples from LCDTC. The first two rows show examples that correctly predict the liquid content of the container by using the two baseline detectors, while the last two rows show examples where the detectors fail to correctly predict it. Note that the number before the line indicates the predicted container state, i.e., e, l, h, m, and f denote ‘empty’, ‘little’, ‘half’, ‘much, and ‘full’, respectively. GT stands for ground truth.

**Qualitative Evaluation.** Some qualitative detection results of the two baseline detectors, i.e., LCD-YOLOF and LCD-YOLOX, are displayed in Figure 8. As shown in the figure, the first two rows display 10 correct prediction samples, while the samples in the last two rows demonstrate instances where the detectors fail to accurately predict the liquid content. The red and purple bounding boxes indicate the detection results of the LCD-YOLOF and LCD-YOLOX, respectively. For the number on the boxes, e.g., e|0.75, the letter before the vertical line denotes the predicted liquid content category (i.e., e, l, h, m, and f represent ‘empty’, ‘little’, ‘half’, ‘much’, and ‘full’, respectively), and the decimal number after the vertical line denotes the predicted score. The first two rows are basically high-quality images with clear texture features and distinct color contrasts against the background. As a result, the two baseline detectors made accurate predictions on these images. However, the liquid in the last two rows tends to display color interference, and the containers have a nonstandard size, making it challenging to correctly detect their liquid content. Consider the last row as an example. From left to right, the opaque container in the first sample is mistakenly identified as a transparent container, likely because of its resemblance to the container’s shape and the object’s small size. The second and third samples illustrate both false and missed detection, stemming from occlusion and the small object size, ultimately leading to inaccurate detection. The fourth and fifth samples display color interference and an absence of distinct texture features, causing the detectors to incorrectly identify the liquid content state. These results indicate that in difficult situations, both LCD-YOLOF and LCD-YOLOX detectors are susceptible to inaccurately identifying the liquid content within transparent containers.

### 5.3. Ablation Study

**Figure 9:**
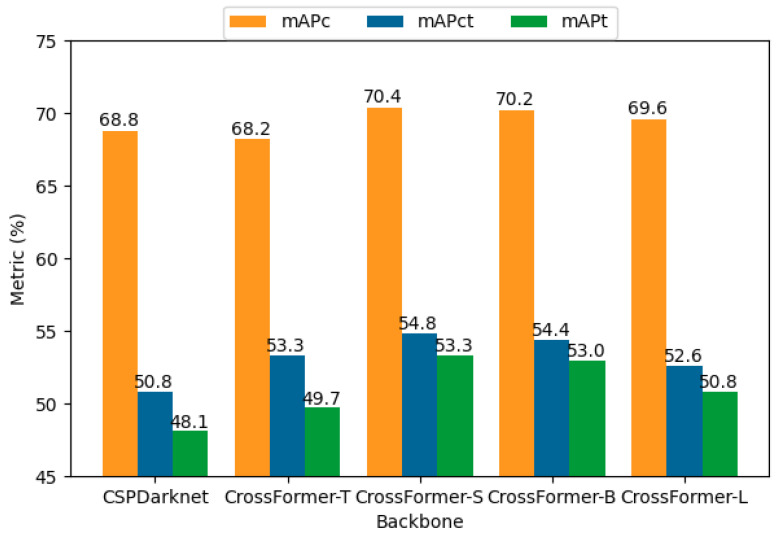
A bar chart depicts the effect of the backbone network on the mAP measure for the LCDTC dataset.

**Impact of the backbone network on LCD-YOLOX.** In order to study the influence of the backbone network of LCD-YOLOX on detecting liquid content in transparent containers, we evaluate LCD-YOLOX with different sizes of backbone networks on LCDTC. Specifically, the backbone network is the CrossFormer with four different variants (-T, -S, -B, and -L stands for tiny, small, base, and large, respectively). For more detailed configurations of CrossFormer’s four variants, please refer to [27]. For comparison we also show the performance with the origin backbone network (i.e., CSPDarknet). Table 3 show the mAPs and the APs at fixed IoUs (i.e., 0.5 and 0.75) with respect to different backbone networks, respectively. As can be seen, with backbone CrossFormer-S, LCD-YOLOX performs best overall except that its $AP_{c}$s are slightly lower than CrossFormer-L, with the highest $mAP_{c}$, $mAP_{t}$, and $mAP_{c}t$ of 0.704, 0.533, and 0.548, respectively. In our paper, we use CrossFormer-S as the default backbone network. In Figure 9, we draw the results of the mAP metric by the bar chart, which provides a more intuitive comparison. We can observe that when the size changes from CSPDarknet to CrossFormer-S, all APs and mAPs consistently improved. However, most of the APs and mAPs decrease with fluctuations when the size ascends from CrossFormer-S to CrossFormer-L, except the $AP_{c}$s, which slightly increase during this process. This can be attributed to the fact that expanding the backbone network’s size requires a greater volume of training data to optimize parameters. Considering the size of our dataset, CrossFormer-S serves as the optimal choice for fine-tuning the proposed task.

**Impact of the proposed components.** To investigate the impact of the proposed components on predicting liquid content state, we train and evaluate the two proposed baseline detectors with and without the proposed components on LCDTC. Table 4 displays the mAPs and APs at fixed IoUs (i.e., 0.5 and 0.75) of two baseline detectors on LCDTC with respect to different proposed components. As can be seen, with the triplet attention incorporated, both LCD-YOLOF and LCD-YOLOX improve their performance overall. Specifically, LCD-YOLOF’s $mAP_{c}$, $mAP_{t}$, and $mAP_{ct}$ increase by 0.5%, 2.7%, and 2.0%, respectively. LCD-YOLOX’s $mAP_{c}$, $mAP_{t}$ and $mAP_{ct}$ increase by 0.3%, 3.0%, and 2.6%, respectively. LCD-YOLOX incorporating CrossFormer also improves its performance overall, with 0.5%, 0.8%, and 1.0% improvements in $mAP_{c}$, $mAP_{t}$, and $mAP_{ct}$, respectively. The observed improvements in mAPs when incorporating triplet attention into the models indeed justify the effectiveness of exploiting triplet attention for enhancing their performance. When LCD-YOLOX incorporates CrossFormer and triplet attention separately or simultaneously, its mAPs increase overall to different extents. For example, with CrossFormer incorporated, LCD-YOLOX’s $AP_{t}$@0.5, $AP_{ct}$@0.75, and $mAP_{ct}$ increase by 2.8%, 3.0%, and 1.0%, respectively. With triplet attention incorporated, its $AP_{t}$@0.5, $AP_{ct}$@0.75, and $mAP_{t}$@0.5 increase by 3.4%, 2.8%, and 3.0%, respectively. With both components incorporated, LCD-YOLOX shows the largest overall improvements, with $mAP_{c}$, $mAP_{t}$, and $mAP_{ct}$ increasing by 1.6%, 5.2%, and 4.0%, respectively. This demonstrates the effectiveness of combining CrossFormer and triplet attention in enhancing the performance of the LCD-YOLOX model. Experimental results suggest that the incorporation of triplet attention in the additional classification head has the potential to enhance the performance of two baseline detectors, whereas in the case of LCD-YOLOX, the utilization of CrossFormer as the backbone network and the introduction of triplet attention in the head would yield noteworthy improvements. That may be explained by the fact that (1) CrossFormer is built on the interactions among features of different scales; (2) PANet, an enhancement built upon FPN’s foundation, also relies on the ability to fuse multiple layers of features, which is one of the foundations for the success of FPN; and (3) triplet attention is able to capture cross-dimensional interactions and provides rich feature representations in order to boost liquid content estimation accuracy.

**Figure 10:**
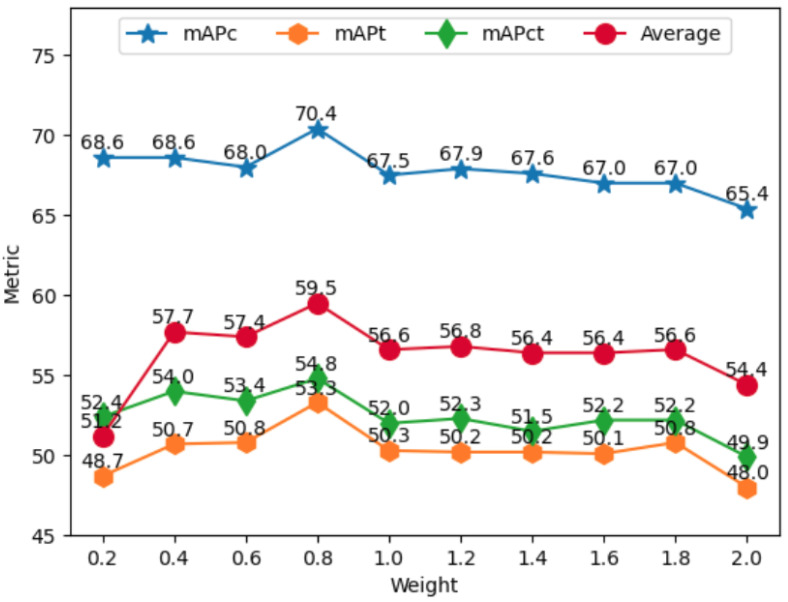
The changing of weighting coefficient for the loss of predicting container state on the LCDTC dataset affect to the values of the three indicators.

**Weighting the loss of predicting liquid content.** In order to study the influence of the weighting coefficient for the loss of predicting liquid content, we evaluate LCD-YOLOX on LCDTC with regard to the weighting coefficient, i.e., λ in Equation (3), which ranges from 0.2 to 2.0 with 0.2 increments. Table 5 display the mAPs and APs of LCD-YOLOX with fixed IoUs (i.e., 0.5 and 0.75) on the LCDTC dataset. Figure 10 show the results of the mAP metric by line chart, facilitating a more intuitive comparative analysis. As presented in Table 5, the best AP values occur within the range from 0.4 to 1.4. However, it is also noted that there are no simultaneous best APs at a fixed λ in LCD-YOLOX. Consequently, it is important to carefully explore and select the appropriate λ value to maximize the AP and overall performance of LCD-YOLOX. We can also observe that the variation between the $AP_{c}$ is basically less than 1%, which indicates that there is little impact on the $AP_{c}$ from the changes in λ, but both $AP_{t}$ and $AP_{ct}$ are greatly affected as λ varies, and the trend of change is basically consistent, denoting that $AP_{t}$ is closely associated with $AP_{ct}$. We can see that the overall maximum values in $AP_{t}$ and $AP_{ct}$ occur at λ = 0.8, which is the default setting in our paper. The experimental results indicate that the maximum values of $AP_{c}@0.5$ and $AP_{ct}@0.5$ are achieved at λ = 1.4 and λ = 0.4, respectively, while other APs demonstrate their best performance when λ is set to 0.8. The results indicate that there may exist a counteracting effect between the localization of transparent container and the estimation of liquid content when these tasks are performed simultaneously as a composite task. Therefore, there is a need to develop more effective approaches that can diminish this counteracting effect, which is a crucial factor that will be taken into account in our future work.

## 6. Conclusions

In this paper, we propose a novel task for identifying liquid content in transparent containers that involves transparent container detection and liquid content estimation. The primary objective of this task is to obtain more information beyond the location of the container by additionally providing certain liquid content information which is easy to achieve with computer vision methods in various open-world applications. To this end, we present the LCDTC dataset which contains 5916 images of transparent containers and establish two baseline detectors, namely LCD-YOLOF and LCD-YOLOX, on the basis of two identity-preserved human posture detectors. By providing the LCDTC dataset and the baseline detectors, we aim to encourage the research community to develop and evaluate new methods and techniques for the challenging task of localizing transparent containers and estimating liquid content. The dataset, along with the baseline detectors, serves as a foundation for future work in this area and can help drive advancements in object detection, particularly for transparent objects.

We anticipate that our work will garner increased interest within the computer vision community, given that liquid content detection in transparent containers is essential for numerous advanced applications, including service robots, pouring robots, security checks, industrial monitoring systems, and beyond. However, it should be noted that the performance of the proposed network architectures is still not satisfying. In our future endeavors, we aim to explore improved techniques to mitigate the counteracting effect between the tasks of localizing transparent containers and identifying their contents, ultimately enhancing the overall performance.

## Figures and Tables

**Table 1:** Comparsion of the APs of the proposed two baseline detectors on the LCDTC dataset. It should be noted that the precision metric for predicting the object’s category, liquid content, and the combination of both are denoted as $AP_{c}$, $AP_{t}$, and $AP_{ct}$, respectively. A bold display is used to highlight the best AP value.

Method	$\{{\mathrm{AP}}_{c},{\mathrm{AP}}_{t},{\mathrm{AP}}_{\mathrm{ct}}\}@0.5$	$\{{\mathrm{AP}}_{c},{\mathrm{AP}}_{t},{\mathrm{AP}}_{\mathrm{ct}}\}@0.75$	$\left\{{\mathrm{mAP}}_{c},{\mathrm{mAP}}_{t},{\mathrm{mAP}}_{\mathrm{ct}}\right\}$
LCD-YOLOF	(0.788,0.553,0.626)	(0.753,0.534,0.603)	(0.660,0.469,0.532)
LCD-YOLOX	(**0.809,0.607,0.624**)	(**0.776,0.588,0.604**)	(**0.704,0.533,0.548**)

**Table 2:** An analysis of the $mAP_{ct}$ difference between the two baseline detectors on the LCDTC dataset has been provided. The mean average precision for the prediction of the combination of the container category and its liquid contents is indicated by the composite $mAP_{ct}$, which should be noted. A bold display is used to highlight the best AP value.

	Empty	Little	Half	Much	Full
$mAP_{ct}$(LCD-YOLOF)	0.474	0.517	0.447	0.474	0.432
$mAP_{ct}$(LCD-YOLOX)	**0.541**	**0.589**	**0.486**	**0.510**	**0.537**

**Table 3:** An analysis was conducted to examine the variations in AP metrics of LCD-YOLOX in relation to varying backbone network sizes on the LCDTC dataset. A bold display is used to highlight the best AP value.

Backbone	$\{{\mathrm{AP}}_{c},{\mathrm{AP}}_{t},{\mathrm{AP}}_{\mathrm{ct}}\}@0.5$	$\{{\mathrm{AP}}_{c},{\mathrm{AP}}_{t},{\mathrm{AP}}_{\mathrm{ct}}\}@0.75$	$\left\{{\mathrm{mAP}}_{c},{\mathrm{mAP}}_{t},{\mathrm{mAP}}_{\mathrm{ct}}\right\}$
CSPDarknet	(0.798,0.552,0.583)	(0.763, 0.534,0.562)	(0.688,0.481,0.508)
CrossFormer-T	(0.808,0.602, 0.617)	(0.775,0.582,0.598)	(0.698,0.529,0.543)
CrossFormer-S	(0.809,**0.607,0.624**)	(0.776,**0.588,0.604**)	(**0.704,0.533,0.548**)
CrossFormer-B	(0.808,0.604,0.619)	(0.776,0.583,0.597)	(0.702,0.530,0.544)
CrossFormer-L	(**0.818**,0.593,0.614)	(**0.784**,0.571,0.591)	(0.696,0.508,0.526)

**Table 4:** Illustration of how the AP metrics of two baseline detectors change with different proposed components on the LCDTC dataset. CF and TA represent CrossFormer and Triplet Attention, respectively. A bold display is used to highlight the best AP value.

Method	CF	TA	$\{{\mathrm{AP}}_{c},{\mathrm{AP}}_{t},{\mathrm{AP}}_{\mathrm{ct}}\}@0.5$	$\{{\mathrm{AP}}_{c},{\mathrm{AP}}_{t},{\mathrm{AP}}_{\mathrm{ct}}\}@0.75$	$\left\{{\mathrm{mAP}}_{c},{\mathrm{mAP}}_{t},{\mathrm{mAP}}_{\mathrm{ct}}\right\}$
LCD-YOLOF	✕	✕	(0.788,0.522,0.602)	(0.751,0.506,0.585)	(0.655,0.442,0.512)
LCD-YOLOF	✕	✓	(0.788,0.553,0.626)	(0.753,0.534,0.603)	(0.660,0.469,0.532)
LCD-YOLOX	✕	✕	(0.798,0.552,0.583)	(0.763, 0.534,0.562)	(0.688,0.481,0.508)
LCD-YOLOX	✓	✕	(0.807,0.580,0.616)	(0.762, 0.557,0.592)	(0.693,0.489,0.518)
LCD-YOLOX	✕	✓	(0.798,0.586,0.613)	(0.763, 0.561,0.590)	(0.691,0.511,0.534)
LCD-YOLOX	✓	✓	(**0.809,0.607,0.624**)	(**0.776,0.588,0.604**)	(**0.704,0.533,0.548**)

**Table 5:** A demonstration of how the weighting coefficient for the loss of liquid content detection in transparent containers alters the AP metrics of LCD-YOLOX on the LCDTC dataset. A bold display is used to highlight the best AP value.

λ	$\{{\mathrm{AP}}_{c},{\mathrm{AP}}_{t},{\mathrm{AP}}_{\mathrm{ct}}\}@0.5$	$\{{\mathrm{AP}}_{c},{\mathrm{AP}}_{t},{\mathrm{AP}}_{\mathrm{ct}}\}@0.75$	$\left\{{\mathrm{mAP}}_{c},{\mathrm{mAP}}_{t},{\mathrm{mAP}}_{\mathrm{ct}}\right\}$
0.2	(0.808,0.560,0.610)	(0.765,0.547,0.585)	(0.686,0.487,0.524)
0.4	(0.808,0.591,**0.629**)	(0.765,0.568,0.603)	(0.686,0.507,0.540)
0.6	(0.807,0.595,0.625)	(0.763,0.572,0.601)	(0.680,0.508,0.534)
0.8	(0.809,**0.607**,0.624)	(**0.776,0.588,0.604**)	(**0.704,0.533,0.548**)
1.0	(0.808,0.592,0.611)	(0.765,0.568,0.585)	(0.675,0.503,0.520)
1.2	(0.817,0.596,0.622)	(0.773,0.570,0.593)	(0.679,0.502,0.523)
1.4	(**0.818**,0.597,0.611)	(0.764,0.568,0.583)	(0.676,0.502,0.515)
1.6	(0.807,0.596,0.620)	(0.773,0.574,0.597)	(0.670,0.501,0.522)
1.8	(0.808,0.602,0.617)	(0.764,0.578,0.593)	(0.670,0.508,0.522)
2.0	(0.798,0.576,0.601)	(0.753,0.552,0.570)	(0.654,0.480,0.499)

## Data Availability

The data that support the fingings of this study are available from the corresponding auther upon reasonable request.

## References

[1] Dhulekar P., Gandhe S., Mahajan U.P. (2018). Development of bottle recycling machine using machine learning algorithm. Proceedings of the 2018 International Conference on Advances in Communication and Computing Technology (ICACCT).

[2] Wang J., Guo W., Pan T., Yu H., Duan L., Yang W. (2018). Bottle detection in the wild using low-altitude unmanned aerial vehicles. Proceedings of the 2018 21st International Conference on Information Fusion (FUSION).

[3] Liu L., Pan Z., Lei B. (2017). Learning a rotation invariant detector with rotatable bounding box. arXiv.

[4] Do C., Schubert T., Burgard W. (2016). A probabilistic approach to liquid level detection in cups using an RGB-D camera. Proceedings of the 2016 IEEE/RSJ International Conference on Intelligent Robots and Systems (IROS).

[5] Aoyagi M., Hiraguri T., Ueno T., Okuda M. (2013). Observation of container liquid levels by dynamic heat conduction. Insight-Non-Destr. Test. Cond. Monit..

[6] Schenck C., Fox D. (2017). Towards learning to perceive and reason about liquids. Proceedings of the 2016 International Symposium on Experimental Robotics.

[7] Narasimhan G., Zhang K., Eisner B., Lin X., Held D. (2022). Self-supervised transparent liquid segmentation for robotic pouring. Proceedings of the 2022 International Conference on Robotics and Automation (ICRA).

[8] Wilson J., Sterling A., Lin M.C. (2019). Analyzing liquid pouring sequences via audio-visual neural networks. Proceedings of the 2019 IEEE/RSJ International Conference on Intelligent Robots and Systems (IROS).

[9] Dong C., Takizawa M., Kudoh S., Suehiro T. (2019). Precision pouring into unknown containers by service robots. Proceedings of the 2019 IEEE/RSJ International Conference on Intelligent Robots and Systems (IROS).

[10] Holland J., Kingston L.M., Mccarthy C., Armstrong E., O’dwyer P., Merz F., McConnell M. (2021). Service Robots in the Healthcare Sector. Robotics.

[11] Cui C., Tang J., fei Qiao J., Wang Z., Sun Z. Review of Waste Plastic Bottle Recycling Equipment Research Status. Proceedings of the 2020 39th Chinese Control Conference (CCC).

[12] Fadlil A., Umar R., Sunardi, Nugroho A.S. (2022). Comparison of Machine Learning Approach for Waste Bottle Classification. Emerg. Sci. J..

[13] Itozaki H., Sato-Akaba H. Detection of bottled explosives by near infrared. Proceedings of the Optics and Photonics for Counterterrorism, Crime Fighting and Defence IX; and Optical Materials and Biomaterials in Security and Defence Systems Technology X.

[14] Cordova A. (2022). Technologies for primary screening in aviation security. J. Transp. Secur..

[15] Chakravarthy S., Sharma R., Kasturi R. (2002). Noncontact level sensing technique using computer vision. IEEE Trans. Instrum. Meas..

[16] Wang T.H., Lu M.C., Hsu C.C.J., Chen C.C., Tan J.D. (2009). Liquid-level measurement using a single digital camera. Measurement.

[17] Eppel S., Kachman T. (2014). Computer vision-based recognition of liquid surfaces and phase boundaries in transparent vessels, with emphasis on chemistry applications. arXiv.

[18] Bobovnik G., Mušič T., Kutin J. (2021). Liquid Level Detection in Standard Capacity Measures with Machine Vision. Sensors.

[19] Do H.T., Thi L.P. (2020). Artificial intelligence (AI) application on plastic bottle monitoring in coastal zone. J. Hydrometeorol..

[20] Xie E., Wang W., Wang W., Ding M., Shen C., Luo P. (2020). Segmenting transparent objects in the wild. Proceedings of the Computer Vision–ECCV 2020: 16th European Conference.

[21] Naseer M., Khan S.H., Porikli F.M. (2018). Indoor Scene Understanding in 2.5/3D for Autonomous Agents: A Survey. IEEE Access.

[22] Schenck C., Fox D. (2017). Visual closed-loop control for pouring liquids. Proceedings of the 2017 IEEE International Conference on Robotics and Automation (ICRA).

[23] Li X., Zhao C., Chen Y., Yi S., Li L., Han G. (2022). Research on Intelligent Detection Technology of Transparent Liquid based on Style Transfer. Proceedings of the 2022 8th International Conference on Big Data and Information Analytics (BigDIA).

[24] Narayan Narasimhan G., Zhang K., Eisner B., Lin X., Held D. (2022). Self-supervised Transparent Liquid Segmentation for Robotic Pouring. arXiv.

[25] Kennedy M., Schmeckpeper K., Thakur D., Jiang C., Kumar V., Daniilidis K. (2019). Autonomous precision pouring from unknown containers. IEEE Robot. Autom. Lett..

[26] Misra D., Nalamada T., Arasanipalai A.U., Hou Q. Rotate to attend: Convolutional triplet attention module. Proceedings of the IEEE/CVF Winter Conference on Applications of Computer Vision.

[27] Wang W., Yao L., Chen L., Cai D., He X., Liu W. (2021). CrossFormer: A Versatile Vision Transformer Based on Cross-Scale Attention. arXiv.

[28] Klank U., Carton D., Beetz M. (2011). Transparent object detection and reconstruction on a mobile platform. Proceedings of the 2011 IEEE International Conference on Robotics and Automation.

[29] Lei Z., Ohno K., Tsubota M., Takeuchi E., Tadokoro S. (2011). Transparent object detection using color image and laser reflectance image for mobile manipulator. Proceedings of the 2011 IEEE International Conference on Robotics and Biomimetics.

[30] Rother C., Kolmogorov V., Blake A. (2004). “GrabCut” interactive foreground extraction using iterated graph cuts. ACM Trans. Graph. (TOG).

[31] Osadchy M. Using specularities for recognition. Proceedings of the IEEE International Conference on Computer Vision.

[32] Mchenry K., Ponce J., Forsyth D. Finding glass. Proceedings of the 2005 IEEE Computer Society Conference on Computer Vision and Pattern Recognition (CVPR’05).

[33] Fritz M., Black M.J., Bradski G.R., Karayev S., Darrell T. An Additive Latent Feature Model for Transparent Object Recognition. Proceedings of the Advances in Neural Information Processing Systems 22: 23rd Annual Conference on Neural Information Processing Systems 2009.

[34] Lai P.J., Fuh C.S. (2015). Transparent object detection using regions with convolutional neural network. In Proceedings of the IPPR Conference on Computer Vision, Graphics, and Image Processing.

[35] Uijlings J.R., Van De Sande K.E., Gevers T., Smeulders A.W. (2013). Selective search for object recognition. Int. J. Comput. Vis..

[36] Girshick R., Donahue J., Darrell T., Malik J. Rich feature hierarchies for accurate object detection and semantic segmentation. Proceedings of the IEEE Conference on Computer Vision and Pattern Recognition.

[37] Khaing M.P., Masayuki M. (2019). Transparent object detection using convolutional neural network. Big Data Analysis and Deep Learning Applications, Proceedings of the First International Conference on Big Data Analysis and Deep Learning, Miyazaki, Japan, 14–15 May 2018.

[38] Liu W., Anguelov D., Erhan D., Szegedy C., Reed S., Fu C.Y., Berg A.C. (2016). Ssd: Single shot multibox detector. Proceedings of the Computer Vision–ECCV 2016: 14th European Conference.

[39] Seib V., Barthen A., Marohn P., Paulus D. (2017). Friend or foe: Exploiting sensor failures for transparent object localization and classification. Proceedings of the 2016 International Conference on Robotics and Machine Vision.

[40] Cao Y., Zhang Z., Xie E., Hou Q., Zhao K., Luo X., Tuo J. (2021). FakeMix augmentation improves transparent object detection. arXiv.

[41] Chen L.C., Papandreou G., Schroff F., Adam H. (2017). Rethinking Atrous Convolution for Semantic Image Segmentation. arXiv.

[42] Wang Z., Peng B., Huang Y., Sun G. (2019). Classification for plastic bottles recycling based on image recognition. Waste Manag..

[43] Xiao J., Tang Y., Zhao Y., Yan Y. (2020). Design of Plastic Bottle Image Recognition System Based on Improved YOLOv3. Proceedings of the 2020 5th International Conference on Mechanical, Control and Computer Engineering (ICMCCE).

[44] Akbar F.S.P., Ginting S.Y.P., Wu G.C., Achmad S., Sutoyo R. (2022). Object Detection on Bottles Using the YOLO Algorithm. Proceedings of the 2022 4th International Conference on Cybernetics and Intelligent System (ICORIS).

[45] Ju L., Zou X., Zhang X., Xiong X., Liu X., Zhou L. (2023). An Infusion Containers Detection Method Based on YOLOv4 with Enhanced Image Feature Fusion. Entropy.

[46] Bochkovskiy A., Wang C.Y., Liao H. (2020). YOLOv4: Optimal Speed and Accuracy of Object Detection. arXiv.

[47] Liu S., Huang D., Wang Y. (2019). Learning Spatial Fusion for Single-Shot Object Detection. arXiv.

[48] Hou Q., Zhou D., Feng J. (2021). Coordinate Attention for Efficient Mobile Network Design. arXiv.

[49] Zhang Y.F., Ren W., Zhang Z., Jia Z., Wang L., Tan T. (2021). Focal and Efficient IOU Loss for Accurate Bounding Box Regression. arXiv.

[50] Feng F., Wang L., Tan M., Yu Z. (2017). Liquid surface location of transparent container based on visual analysis. Proceedings of the 2017 First International Conference on Electronics Instrumentation & Information Systems (EIIS).

[51] Shen J., Castan S. (1992). An optimal linear operator for step edge detection. CVGIP Graph. Model. Image Process..

[52] Feng F., Wang L., Zhang Q., Lin X., Tan M. (2012). Liquid surface location of milk bottle based on digital image processing. Proceedings of the Multimedia and Signal Processing: Second International Conference, CMSP 2012.

[53] Mottaghi R., Schenck C., Fox D., Farhadi A. See the glass half full: Reasoning about liquid containers, their volume and content. Proceedings of the IEEE International Conference on Computer Vision.

[54] Everingham M., Van Gool L., Williams C.K., Winn J., Zisserman A. (2009). The pascal visual object classes (voc) challenge. Int. J. Comput. Vis..

[55] Lin T.Y., Maire M., Belongie S., Hays J., Perona P., Ramanan D., Dollár P., Zitnick C.L. (2014). Microsoft coco: Common objects in context. Proceedings of the Computer Vision–ECCV 2014: 13th European Conference.

[56] Guo Y., Chen Y., Deng J., Li S., Zhou H. (2023). Identity-Preserved Human Posture Detection in Infrared Thermal Images: A Benchmark. Sensors.

[57] Ge Z., Liu S., Wang F., Li Z., Sun J. (2021). Yolox: Exceeding yolo series in 2021. arXiv.

[58] Chen Q., Wang Y., Yang T., Zhang X., Cheng J., Sun J. You only look one-level feature. Proceedings of the IEEE/CVF Conference on Computer Vision and Pattern Recognition.

[59] Liu S., Qi L., Qin H., Shi J., Jia J. Path aggregation network for instance segmentation. Proceedings of the IEEE Conference on Computer Vision and Pattern Recognition.

[60] He K., Zhang X., Ren S., Sun J. Deep residual learning for image recognition. Proceedings of the IEEE Conference on Computer Vision and Pattern Recognition.

[61] Deng J., Dong W., Socher R., Li L.J., Li K., Fei-Fei L. (2009). Imagenet: A large-scale hierarchical image database. Proceedings of the 2009 IEEE Conference on Computer Vision and Pattern Recognition.

[62] Qin L., Zhou H., Wang Z., Deng J., Liao Y., Li S. (2022). Detection Beyond What and Where: A Benchmark for Detecting Occlusion State. Proceedings of the Pattern Recognition and Computer Vision: 5th Chinese Conference, PRCV 2022.

[63] Lin T.Y., Goyal P., Girshick R., He K., Dollár P. (2017). Focal Loss for Dense Object Detection. IEEE Trans. Pattern Anal. Mach. Intell..

[64] He K., Zhang X., Ren S., Sun J. (2015). Spatial pyramid pooling in deep convolutional networks for visual recognition. IEEE Trans. Pattern Anal. Mach. Intell..

[65] Liu Z., Lin Y., Cao Y., Hu H., Wei Y., Zhang Z., Lin S., Guo B. Swin transformer: Hierarchical vision transformer using shifted windows. Proceedings of the IEEE/CVF International Conference on Computer Vision.

[66] Lin H., Cheng X., Wu X., Shen D. (2022). Cat: Cross attention in vision transformer. Proceedings of the 2022 IEEE International Conference on Multimedia and Expo (ICME).

[67] Wang W., Xie E., Li X., Fan D.P., Song K., Liang D., Lu T., Luo P., Shao L. Pyramid vision transformer: A versatile backbone for dense prediction without convolutions. Proceedings of the IEEE/CVF International Conference on Computer Vision.

